# The Impact of Epidemics and Pandemics on the Mental Health of Healthcare Workers: A Systematic Review

**DOI:** 10.3390/ijerph18136695

**Published:** 2021-06-22

**Authors:** Ottilia Cassandra Chigwedere, Anvar Sadath, Zubair Kabir, Ella Arensman

**Affiliations:** 1 School of Public Health, University College Cork, T12 XF62 Cork, Ireland; anvar.sadathvakkayil@ucc.ie (A.S.); z.kabir@ucc.ie (Z.K.); ella.arensman@ucc.ie (E.A.); 2 National Suicide Research Foundation, University College Cork, T12 XF62 Cork, Ireland; 3 Australian Institute for Suicide Research and Prevention, Griffith University, Brisbane, QLD 4059, Australia

**Keywords:** epidemics and pandemics, mental health and healthcare workers, COVID-19

## Abstract

Background: There is increasing evidence that healthcare workers (HCWs) experience significant psychological distress during an epidemic or pandemic. Considering the increase in emerging infectious diseases and the ongoing COVID-19 pandemic, it is timely to review and synthesize the available evidence on the psychological impact of disease outbreaks on HCWs. Thus, we conducted a systematic review to examine the impact of epidemics and pandemics on the mental health of HCWs. Method: PubMed, PsycInfo, and PsycArticles databases were systematically searched from inception to June-end 2020 for studies reporting the impact of a pandemic/epidemic on the mental health of HCWs. Results: Seventy-six studies were included in this review. Of these, 34 (45%) focused on SARS, 28 (37%) on COVID-19, seven (9%) on MERS, four (5%) on Ebola, two (3%) on H1N1, and one (1%) on H7N9. Most studies were cross-sectional (93%) and were conducted in a hospital setting (95%). Common mental health symptoms identified by this review were acute stress disorder, depression, anxiety, insomnia, burnout, and post-traumatic stress disorder. The associated risk factors were working in high-risk environments (frontline), being female, being a nurse, lack of adequate personal protective equipment, longer shifts, lack of knowledge of the virus, inadequate training, less years of experience in healthcare, lack of social support, and a history of quarantine. Conclusion: HCWs working in the frontline during epidemics and pandemics experience a wide range of mental health symptoms. It is imperative that adequate psychological support be provided to HCWs during and after these extraordinary distressful events.

## 1. Introduction

The frequency of disease outbreaks has increased over the past century due to population growth, the increased interconnectedness of the world, microbial adaptation and change, economic development, changes in land use, and climate change [1]. Emerging infectious diseases that have caused epidemics over the past two decades include the severe acute respiratory syndrome (SARS) in 2003, Influenza A virus subtype H1N1 in 2009, Middle East respiratory syndrome coronavirus (MERS-COV) in 2012, Ebola Virus Disease (EVD) in 2014, the influenza A virus subtype H7N9, and the severe acute respiratory syndrome coronavirus 2 (SARS-CoV-2) in December 2019, which has resulted in the coronavirus disease 2019 (COVID-19) pandemic [2].

Disease outbreaks cause an unexpected increase in morbidity and mortality, which in turn cause an increased demand on healthcare facilities [3]. The rapid increase in patient populations drastically reduces the healthcare worker (HCW) to patient ratio thus increasing workload. HCWs suffer from both physical and mental fatigue because their working hours are increased and they may be asked to work more night shifts; thus, they do not have enough time to sleep, rest, and recuperate. As they work in the frontline, diagnosing, managing, and caring for sick patients, they experience a variety of mental health symptoms which may also persist after the epidemic has ended [4].

The massive influx of patients overwhelms the capacity of healthcare systems, giving rise to ethical dilemmas around the distribution of essential healthcare and medical supplies. HCWs constantly have to make “life or death” decisions, such as which patients to admit or not admit into intensive care and when to withdraw life support [5]. Due to the increased numbers of people dying, HCWs repeatedly break bad news, sometimes in ways they are not used to, including over the phone, thus making breaking bad news more distressing [6]. The news of continuously rising numbers of confirmed cases and deaths is emotionally overwhelming.

The shortage in supplies of personal protective equipment (PPE) may increase the risk and fear of contagion [7]. During this time, HCWs continuously live with anxiety and fear of contracting the disease more so when a colleague becomes infected or dies [8]. They fear transmitting the infection to their families as well as experiencing stigma and discrimination from their communities due to transmission fears. This type of stigmatization may even escalate to harassment, being denied access to public transport, physical violence, and eviction from their homes by landlords [9]. Social ostracization aggravates the occupational stress that HCWs are already facing as they battle the disease outbreak [10].

Overall, these negative psychological factors do not only affect the HCWs themselves, but also reduce their effectiveness in fighting epidemics, therefore indirectly affecting the whole population at large. This systematic review aims to synthesize the available evidence on the impact of epidemics and pandemics on the mental health of HCWs which will guide and inform best practice policies for psychological supports and mental health interventions for HCWs. Even though similar systematic reviews have been conducted recently [11,12,13,14,15,16], these were either specific to a single pandemic [11] (i.e., SARS) or Covid-19 [13,14,15,16], or with a small number of studies [12,13,16], and included only one study from low- and middle-income countries [12].

## 2. Materials and Methods

This systematic review followed the Preferred Reporting Items for Systematic Reviews and Meta-Analysis Protocols guidelines (PRISMA) [17]. However, the review is descriptive in nature, and the data extracted from the selected studies were summarized but not statistically combined owing to methodological heterogeneity. The study protocol was pre-registered with the National Institute for Health Research international prospective register of scientific reviews (PROSPERO, CRD42020186331) [18].

### 2.1. Data Sources and Search Strategy

A comprehensive systematic search of the most common databases—PubMed, PsycInfo, and PsycArticles, was conducted from inception to June-end 2020. Furthermore, the reference list of the retrieved articles and systematic reviews of similar topics were also examined to verify whether any potential studies had been left out. The author (O.C.C.) conducted the initial literature search. The full search strategy is available in Appendix A. Combinations of the following terms were used for the search:

Category 1: Population (healthcare professional, healthcare workers, physician, doctor, nurse)

Category 2: Exposure (epidemic, pandemic, SARS, MERS, Ebola, H1N1, H7N9, COVID 19). The terms epidemic and pandemic were defined according to the World Health Organization definitions. “An epidemic is the occurrence in a community or region of cases of an illness, specific health-related behavior, or other health-related events clearly in excess of normal expectancy” [19]. “A pandemic is the worldwide spread of a new disease” [20].

Category 3: Outcomes (mental health, mental disorder, psychological, depression, anxiety, stress, burden, insomnia, sleep disturbance, burnout, fear, stigma, discrimination).

Search results citations were downloaded to Endnote reference management software version X9 and duplicates were removed. The author (O.C.C.) performed the initial screening of titles and abstracts for relevance.

### 2.2. Eligibility Criteria

The following criteria were applied for papers to be included in the review:

1. Studies reporting the impact of a pandemic/epidemic on mental health outcomes of health care workers.
2. Cross-sectional, case–control, and cohort studies. Intervention studies were considered for inclusion only when they had sufficient details about the baseline mental health outcomes.
3. Studies were selected if data was from an original study
4. Studies had to be published in a peer reviewed journal.
5. Only English language studies were included.
6. No restrictions were placed on the publication date.
7. There was no limit on the geographical location of studies.

Preprints, study protocols, and conference abstracts/proceedings were excluded.

### 2.3. Data Extraction and Quality Appraisal

The author (O.C.C.) checked the relevant studies for eligibility and extracted data from the eligible studies onto a standard Microsoft Excel data extraction form. A second reviewer (A.S.) independently verified the eligibility of the included studies. Any discrepancies were resolved by discussion. Full text articles for eligible studies were obtained. The data extraction form included the author(s) of the study, the publication year, country of study, details about study participants, study settings, study design, outcome measures used, and main findings. Furthermore, information necessary for evaluating study quality was also extracted from the eligible studies. Studies were assessed for methodological quality using the Joanna Briggs Institute (JBI) critical appraisal tools for cross sectional [21] and cohort studies [22].

### 2.4. Data Analysis and Synthesis

The authors analyzed and synthesized the results using a narrative text approach to summarize and explain the study findings focusing on prevalence of mental health outcomes, and the associated risk and protective factors.

## 3. Results

### 3.1. Study Selection Process

**Figure 1:**
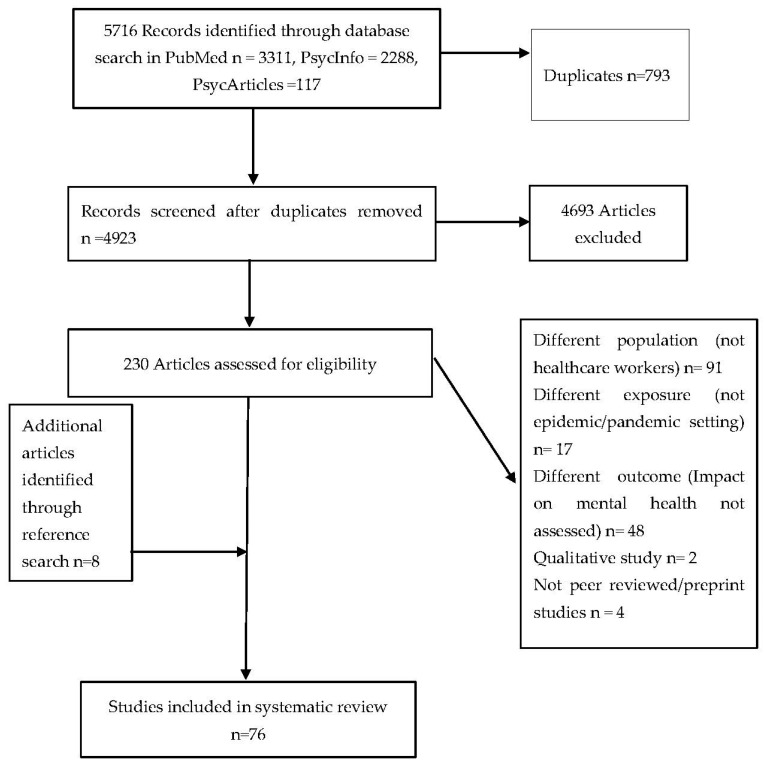
PRISMA flow diagram of studies selected for inclusion in systematic review.

The three database searches yielded 5716 articles. After removal of 793 duplicates, the titles and abstracts of 4923 articles were screened. Two-hundred-and-thirty potential studies were identified, and the full texts were checked for eligibility. Sixty-eight articles met the inclusion criteria and eight more were identified through searching references of selected papers totaling 76 final studies. Details are provided in the PRISMA flowchart (Figure 1).

### 3.2. Characteristics of Selected Studies

The characteristics of the selected studies are shown in Table 1 and Table 2. Overall, seventy-six papers met the inclusion criteria. Of these, 34 (44%) focused on SARS, 28 (37%) on COVID-19, seven (9%) on MERS, four (5%) on Ebola, 2 (3%) on H1N1, and one (1%) on H7N9. The studies were conducted in different countries: 26 (34%) China; nine (12%) Taiwan; seven (9%) Canada; eight (11%) Hong Kong; seven (9%) Singapore; four (5%) Saudi Arabia; four (5%) Korea; one (1%) each from Germany, Greece, Iran, Italy, Japan, Liberia, Sierra Leonne, Nigeria, Turkey, and USA; and one was conducted in two countries Singapore and India. Most studies were conducted in a hospital setting 71 (95%), three in a general practice setting, and one at a rehabilitation center. Sixty studies (80%) included more than one type of HCW, 12 had only nurses, and three had only doctors/physicians. A higher proportion of studies 71 (93%) studies were cross-sectional and only 5 (7%) were cohort studies.

### 3.3. Quality Appraisal

A more detailed assessment is available in Table 3 and Table 4. All eligible studies were included in the review, regardless of their quality assessment results. Of the 71 cross-sectional studies, 42 papers (59%) were of very good quality, five papers (7%) were of good quality, 15 papers (21%) were of average quality, and nine papers (13%) were of poor quality. Of the five cohort studies, one paper was of very good quality, two papers were of good quality, one paper had average quality, and one was of poor quality.

### 3.4. Commonly Used Mental Health Instruments in This Analysis

The Impact of Events Scale (IES) and the Perceived Stress Scale (PSS) were the most common instruments used to measure stress. The Generalized Anxiety Disorder (GAD) and the Zung Self-Rating Anxiety Scale (SAS) were frequently used instruments to measure anxiety. Commonly used instruments to measure depression were the Patient Health Questionnaire (PHQ) and the Zung Self-Rating Depression Scale. Insomnia was often measured using the Insomnia Severity Index (ISI) and the Pittsburgh Sleep Quality Index (PSQI). Most studies which measured burnout used the Maslach’s Burnout Inventory.

### 3.5. Mental Health Findings

#### 3.5.1. Stress

Stress was the most commonly measured mental health symptom. Any one of acute stress, distress, or post-traumatic stress symptoms was examined in forty-two studies [4,24,25,26,29,30,35,38,40,44,45,49,52,53,54,56,57,58,60,62,63,64,73,74,75,76,78,80,81,82,83,84,86,87,89,90,91,92,95,96,97]. The prevalence of stress varied, and it ranged from 5% to 80%. Ten studies identified that nurses experienced more distress compared to doctors [30,38,54,63,64,80,81,82,87,91]. HCWs providing direct care to confirmed cases of SARS and COVID 19 were more likely to be distressed compared to those who did not provide direct care [30,45,53,58,63,76,78,91,92]. Moving from a low risk ward to work in a high risk ward [75], more working time per week [35], frequent changes in infection control measures and protocols [79], seeing a colleague getting sick, being intubated or dying increased stress [57] while those who received adequate social support were least likely to have PTSD [90]. Having been in quarantine during the outbreak was associated with high levels of PTSD [4,62,83]. Availability of adequate PPE significantly reduced stress [38,49,90].

#### 3.5.2. Anxiety and Fear

Anxiety and fear symptoms were examined in 29 studies [23,25,26,27,28,29,30,32,33,34,35,36,37,40,41,42,44,45,46,48,50,51,59,64,68,88,93,96,97]. The prevalence of anxiety varied and ranged from 7% to 78% across all virus exposures. Nine studies found that HCWs who had contact with confirmed cases had more anxiety compared to HCWs who had had no contact with confirmed cases [27,30,33,34,37,44,88,97]. A common cause of anxiety was worrying about transmitting infection to family members [41,51,88]. Nurses had higher anxiety scores compared to doctors [27,30,35,37,38,50,88]. Female healthcare workers were more likely to have anxiety compared to males [26,27,30,45,46,48,56,88]. Three studies from China compared anxiety levels of HCWs in Wuhan to those of HCWs in the outreach or other regions and found that HCWs in Wuhan, which was the epicenter of COVID-19 at that time, had significantly higher anxiety compared to HCWs in other regions of China [26,30,33]. Similar results were found in Canada were HCWs in Toronto who had more contact with SARS patients had higher levels of burnout and distress compared to HCWs in Hamilton where they had fewer confirmed cases [73]. Fear and anxiety were significantly increased when a colleague became infected or died. Anxiety and fear of infection were inversely related to availability of hospital resources, HCWs’ resilience and support from family and friends [26,28]. The increase in working hours during a disease outbreak was directly related to anxiety levels [27,35]. Lack of knowledge of the virus was also associated with an increase in anxiety [59].

#### 3.5.3. Depression

Symptoms of depression were examined in 25 studies [23,25,26,28,29,30,32,34,36,37,38,40,42,44,45,46,48,49,50,59,62,67,71,96,97]. The prevalence of depression ranged from 8.9% and 74.2%. Five studies showed that depression was higher in females compared to males [30,45,46,49,50]. The frontline medical staff working in the respiratory, emergency, ICU, and infectious disease departments were twice more likely to suffer from depression than the non-clinical staff [30,34,44]. Nurses working in SARS units were more depressed than nurses in non-SARS units [97]. The HCWs in Wuhan, which was the epicenter of the COVID-19 pandemic, had higher levels of depression compared to HCWs outside Hubei province [26,30]. Increased working hours were associated with elevated depression and hopelessness [27,35]. Having a past exposure to traumatic events or pre-existing psychiatric disorder before the epidemic was associated with high levels of depressive symptoms [62,95]. Those HCWs with a marital status of being single were more likely than married HCWs to have high levels of depressive symptoms [46,62]. A history of being quarantined was associated with higher levels of depression [62]. Support from family and friends [26,28,34], psychological preparedness, altruistic acceptance, and perceived efficacy of dealing with the pandemic was associated with lower levels of depression [46,62].

#### 3.5.4. Insomnia and Sleep Quality

Insomnia and sleep quality was assessed in 11 studies [23,29,30,36,37,38,42,44,48,71,97]. All 11 studies reported substantial sleep problems, ranging from 26% to 45%. Insomnia was independently associated with depression and anxiety [23,42]. In three studies, insomnia symptoms were higher in frontline HCWs compared to second line workers [30,36,37,42]. Nurses reported more sleep problems compared to other HCWs [30,37,38], and nurses working in SARS units were more likely to have insomnia compared to nurses working in non-SARS units [97]. HCWs in Wuhan reported more insomnia symptoms compared to healthcare workers in other areas out of Hubei province [30].

#### 3.5.5. Burnout (Emotional Exhaustion)

Burnout (emotional exhaustion) was assessed in eight studies, and they all confirmed high levels of burnout in HCWs [28,43,58,70,72,73,88,96]. HCWs who worked in the frontline or had contact with confirmed cases were more likely to be emotionally exhausted compared to HCWs who were not in the frontline and who had no direct contact with confirmed cases [70,72,73,88], while one study reported different results in that front-line HCWs had lower levels of burnout compared to other HCWs. The possible explanation given by the researchers for this unexpected trend was front-line HCWs had received timely and accurate information hence they had a higher sense of control of their situation [43]. Two studies showed that HCWs who had spent more time in quarantine had higher levels of burnout [70]. Lower levels of organizational support, job stress and poor hospital resources, were directly related to emotional exhaustion [58,70,72]. Burnout was negatively correlated to self-efficacy, resilience and family support [28]. High anxiety scores predicted high levels of burnout [88].

#### 3.5.6. Stigma

Five studies examined stigma and in all studies, HCWs had been stigmatized either by their family or by the community or both [52,76,78,83,92]. The prevalence of stigma in HCWs ranged from 20% to 49%. HCWs who were working in direct contact with confirmed cases and those who had been quarantined experienced higher levels of stigma [76,92]. One study which compared psychological morbidity of stigma between general practitioners and Chinese traditional practitioners found that general practitioners had more exposure to SARS patients and suffered more stigma than the Chinese traditional practitioners [92]

## 4. Discussion

This review showed that epidemics and pandemics have a negative impact on the psychological wellbeing of HCWs by the wide range of mental health symptoms, in particular stress, depression, anxiety, insomnia, fear, stigma, and emotional exhaustion.

This review identified common factors that increased the risk of mental health symptoms. Frontline HCWs working in high risk environments where they had direct contact with suspected and confirmed cases of SARS and COVID 19 reported more psychological symptoms compared to non-frontline HCWs working in low risk environments [30,31,34,36,37,43,44,45,48,53,58,63,65,66,69,73,75,76,85,91,92,96]. Working in direct contact with infectious patients was associated with higher levels of symptoms of anxiety, stress, insomnia, and depression due to the increased fear of contracting infection, greater concern of infecting family members, stigmatization, and isolation [34,54,72,88]. This might explain why nurses were found to be more stressed, anxious, depressed, and had poorer sleep quality compared to doctors. Most studies explained this to be due to the higher workload that nurses have and the more time they spend in direct contact with patients whilst nursing them [27,30,37,38,41,50,54,63,72,76,80,81,82,87,88,91]. HCWs in the epicenter of a pandemic experienced more psychological distress compared to HCWs in other regions due to the higher exposure to infectious patients [26,30,33,73]. Another occupational risk factor identified was the extent of healthcare experience that a HCW had. HCWs with less work experience were more likely to be stressed compared to HCWs with more years of work experience. Less experienced HCWs have less knowledge, skills, and are less able to self-regulate, thus they get stressed more easily compared to more experienced HCWs who have more knowledge and skills, and are thus more able to adapt [53,54,96].

Inadequate hospital equipment and the limited supply of personal protective equipment (PPE) were also associated with higher levels of psychological symptoms [23,34,38,58]. Being of female gender was also identified as a risk factor [27,29,30,38,39,45,48,49,50,54,56,62,81,85,91]. A history of exposure to other traumatic events before an t outbreak increased the risk of re-occurrence of a psychiatric disorder [62,95]. Having a high perceived risk of infection and low self-efficacy were also identified as risk factors associated with mental health symptoms [49,56,62,74,87]. HCWs who were unconfident about beating the outbreak [49,56,62,74,87] were more depressed and had a poor mental state compared to HCWs who were more confident and resilient [28,77]. Lack of knowledge of the virus and lack of outbreak management training was associated with low perceived self-efficacy. Constantly changing infection control measures and documentation processes also reduced self-efficacy and caused an increase in stress levels [45]. Having been quarantined was identified as a risk factor of depressive and post-traumatic stress symptoms. This was attributed to the increased fear of dying from the disease. Quarantining was associated with increased levels of fear and stress in HCWs due to the emotional isolation and loneliness experienced during quarantine [39,62,65,67,70,77,83].

Despite the limited number of cohort studies compared to cross sectional studies, the cohort studies conducted during the SARS epidemic confirmed the persistence of mental health symptoms up to a year after the pandemic has ended.

### 4.1. Protective Factors

Protective factors identified in this systematic review include adequate information, clear guidelines, training and organizational support [24,43,70,71,72,78,79,95], altruistic acceptance of risk, [62,65], availability of specialized equipment for treating patients, adequate personal protective equipment [49,57,74,78,90], having more years of healthcare experience [95], adequate time off work [68], and support from family and friends [71,90].

### 4.2. Strengths and Limitations of This Review

The strengths of this review are, first, that it identified a large number of studies conducted during and after the epidemics and pandemics that have occurred in the past twenty years, including the current COVID-19 pandemic. Second, results are generalizable as the included studies were from Asia, Europe, Africa, Middle East, and America. Third, most papers included in this review used standardized and previously validated instruments for measuring mental health symptoms. However, a potential limitation is that we only included published articles and excluded gray literature, which might have caused some publication bias. Another limitation is that there were only five cohort studies, 94% of the studies included were cross-sectional which implies that no causal inferences can be drawn. Furthermore, meta-analyses were not undertaken because of the methodological heterogeneity of the studies.

### 4.3. Recommendations for Future Research and Mental Health Practice

It is important to conduct more cohort studies to obtain a detailed picture of mental health symptoms at the different points of a disease outbreak, and to understand the long-term mental health impact of a pandemic or epidemic among HCWs.

The possible role of occupation and exposure on mental health needs to be examined further in future studies. While many studies have reported higher levels of mental health problems among female HCWs, it is still unclear whether gender is a sole influencing factor, or if gender is being confounded by other factors. For instance, most of the female HCWs were nurses, and nurses experience higher mental health problems due to their increased exposure and nature of work. Besides, previous studies have shown that nurses and doctors working in the emergency department and intensive care units are at a higher risk of burnout, depression, and job stress compared to their colleagues working in other hospital departments [98,99,100]. Therefore, future studies need to rule out these aspects, while determining the effects of a pandemic or epidemic on mental health.

Increasing age, and prior chronic medical conditions make a person more susceptible to the effects of a pandemic. Therefore, in future studies, it is important to address the association between these factors and mental health outcome.

Many studies used online platforms for data collection, and this method is known to increase the risk of sampling and response bias [101]. However, we consider this method as appropriate for the current studies as face-to-face data collection was not possible due to social distancing guidelines.

As this review identified many protective factors including adequate information about the pandemic, clear guidelines and training, social support, availability of specialized equipment for treating patients, adequate personal protective equipment, adequate time off work, may be provided to the HCWs for reducing adverse mental health outcome.

## 5. Conclusions

This systematic review provides a comprehensive narrative synthesis of the underlying negative impacts of epidemics and pandemics on the mental health of HCWs which include acute stress, post-traumatic stress disorders, severe depression, anxiety, burnout, insomnia, and stigmatization. It is apparent from this review that the current healthcare systems and many governments across the globe need to prioritize mobilizing resources to provide sufficient and necessary psychological support to HCWs during and after epidemics and pandemics.

## Figures and Tables

**Table 1:** A summary of the cross-sectional studies included in this review.

Study (Year)/Country	Disease Outbreak	Participants (Setting)	Mental Health Outcome Measures (Instrument)	Main Findings
Amerio et al. (2020) [23] Italy	COVID-19 Epidemic	N = 131 General Practitioners (General Practice)	Depression (PHQ-9) Anxiety (GAD-7) Insomnia (ISI) Health Related Quality of Life HrQoL (SF-12)	22 9%: PHQ-9 ≥ 10 moderate to severe depression and 77.1%: PHQ-9 ≤ 10 Mild to moderate depression Physicians with moderate to severe depression had higher severity for anxiety and insomnia and poorer HrQoL. Physicians with moderate to severe depression perceived less adequate PPE
Cai et al. (2020) [24] China	COVID-19 Pandemic	N = 534 HCWs (Hospital)	Emotions, factors that increase stress, factors that reduce stress, coping strategies (self-designed questionnaire)	Medical staff were anxious regarding their safety and the safety of their families and reported adverse psychological effects from reports of mortality from COVID-19 infection.
Chew et al. (2020) [25] Singapore and India	COVID-19 Pandemic	N = 906 HCWs. 480 from Singapore and 426 from India (Hospital)	Depression, anxiety, and stress (DASS-21) Psychological distress and PTSD (IES)	15.7% participants had anxiety. 10.6% had depression. 5.2% had stress. There was no difference in psychological outcomes between study participants from the two countries. The presence of physical symptoms was associated with higher mean scores in the IES-R, DASS-21 scales
Du et al. (2020) [26] China	COVID-19 Pandemic	N = 134 HCWs 60 Wuhan vs. 74 Outreach (Hospital)	Depression (BDI-II) Anxiety (BAI) Stress (PSS)	12.4% Depressive symptoms (BDI-II scores ≥ 14) 20.1% Anxiety symptoms (BAI scores ≥ 8) 59.0% moderate to severe stress (PSS scores ≥14) Depression and anxiety higher in Wuhan vs. outreach workers Depression and anxiety higher in females and those having poor family support.
Hacimusalar et al. (2020) [27] Turkey	COVID-19 Pandemic	N = 2156 1121 HCWs vs. 1035 non-HCWs (society/social media)	State Trait Anxiety Scale (STAI) Hopelessness (BHS)	The hopelessness and state anxiety levels of HCWs were higher than non-HCWs. Nurses’ anxiety and hopelessness levels were higher than doctors and other HCWs. Anxiety and hopelessness levels were higher in females, those living with a high-risk individual at home, those with difficulty in caring for their children, those with increased working hours and those whose income decreased
Hu et al. (2020) [28] China	COVID-19 Pandemic	N = 2014 nurses (Hospital)	Burnout (MBI) Anxiety (SAS) Depression (SDS) Fear (FS-HPs)	Burnout: High burnout during work Anxiety: 27.1% Mild, 11.0% Moderate, 3.3% Severe Depression: 32.8% Mild, 9.6% Moderate, 1.1% Severe Fear:28% Moderate, 36.2% High HCWs who had low self-efficacy and did not have family and social support had worse mental health outcomes
Kang et al. (2020) [29] China	COVID-19 Pandemic	N = 994 183 Doctors and 811 Nurses (hospital)	Depression (PHQ-9) Anxiety (GAD-7) Insomnia (ISI) Distress (IES)	36% had subthreshold mental health disturbances (mean PHQ9: 2.4, GAD-7: 1.5, ISI: 2.8, IES-R: 6.1), 34.4% had mild disturbances (mean PHQ-9: 5.4, GAD-7: 4.6, ISI: 6.0, IES-R: 22.9), 22.4% had moderate disturbances (mean PHQ-9: 9.0, GAD-7: 8.2, ISI: 10.4, IES-R: 39.9) 6.2% had severe disturbances (mean PHQ-9: 15.1, GAD-7: 15.1, ISI: 15.6, IES-R: 60.0) Women had more psychological burden than men
Lai et al. (2020) [30] China	COVID-19 Pandemic	N = 1257 764 Nurses 493 Doctors (Hospital)	Depression (PHQ-9) Anxiety (GADS-7) Insomnia (ISI-7) Distress (IES)	50.4% Depression, 44.6% Anxiety, 34.0% Insomnia, 71.5% Distress Nurses had more severe degrees of mental health symptoms than other HCWs Females had worse mental health symptoms compared to men. Frontline HCWS had higher levels of mental health symptoms compared to second line workers. HCWS in Wuhan had more distress compared to HCWs outside Wuhan and outside Hubei province
Li et al. (2020) [31] China	COVID-19 Pandemic	N = 526 nurses and 214 general public 234 frontline nurses 292 non frontline nurses (Hospital)	Vicarious traumatization (Self-developed questionnaire)	Vicarious traumatization scores for non-front-line nurses were significantly higher than those of front-line nurses. Vicarious traumatization scores of the general public were significantly higher than those of the front-line nurses. No significant difference was noted in vicarious traumatization scores between the general public and non-front-line nurse
Liang et al. (2020) [32] China	COVID-19 Pandemic	N = 56 HCWs (Hospital)	Anxiety (SAS) Depression (SDS)	Several staff were experiencing clinically significant depressive and anxiety symptoms.
Liu et al. (2020) [33] China	COVID-19 Pandemic	N = 512 HCWs (Hospital)	Anxiety (SAS)	12.5% Anxiety prevalence Anxiety score was significantly higher among the medical staff treating confirmed cases vs. those who had not. Staff from Hubei province had higher anxiety compared to staff from other parts of China
Lu et al. (2020) [34] China	COVID-19 Pandemic	N = 2299 HCWs (hospital)	Fear (NRS) Anxiety (HAMA) Depression (HAMD)	Medical staff experienced more fear, anxiety, and depression compared to administrative staff. Front line medical staff in direct contact with COVID-19 patients in the respiratory, emergency, infectious disease, and ICU departments had higher scores of fear, anxiety, and depression compared to those who did not have contact with infected patients. Lack of PPE, loneliness from being isolated from family and loved ones worsened anxiety and depression
Mo et al. (2020) [35] China COVID-19 Pandemic	COVID-19 Pandemic	180 nurses (Hospital)	Stress (SOS) Anxiety (SAS)	Nurses’ anxiety scores were significantly higher than the national standard scores (32.19 vs. 29.78) Anxiety scores were positively correlated to total stress load. Being an only child in their family and more working hours a week predicted higher levels of anxiety and stress.
Qi et al. (2020) [36] China	COVID-19 Pandemic	N = 1306 medical workers 801 FMW vs. 505 non-FMW (hospital)	Sleep quality (PSQI) Insomnia (AIS) Anxiety (VAS) Depression (VAS)	FMW had a higher prevalence of sleep disturbances PSQI > 6 compared to non FMW (78.4% vs. 61.0%) FMW had a higher prevalence of sleep disturbances AIS > 6 compared to non FMW (51.7% vs. 31.6%) FMW had higher depression and anxiety scores compared to non FMW
Que et al. (2020) [37] China	COVID-19 Pandemic	N = 2285 HCWs (Hospital)	Anxiety (GAD-7) Depression (PHQ-9) Insomnia (ISI)	56.59% Overall psychological problems 46.04% Anxiety 44.37% Depression 28.75% Insomnia Frontline HCWs had a higher risk of anxiety, insomnia and overall psychological problems compared to non-frontline HCWs. Highest prevalence of anxiety and insomnia was observed in nurses
Shechter et al. (2020) [38] USA	COVID-19 Pandemic	N = 657 HCWs (Hospital)	PTSD (PC-PTSD) Psychological (PHQ-2) Depression (GAD-2) Insomnia (ISI) Sleep quality (PSQI)	57% PTSD symptoms 48% depressive symptoms 33% anxiety symptoms 26% reported severe or very severe sleep problems. Nurses were more likely than attending physicians to screen positive for stress, depression, anxiety, and sleep problems. Lack of national guidelines and lack of adequate PPE were major stressors. 59% Physical exercise was the most common coping behavior. 33% accessed a therapist with online self-guided counselling. Women reported more severe symptoms compared to men
Sun et al. (2020) [39] China	COVID-19 Pandemic	N = 442 HCWs (hospital)	Distress (IES) Distress (2019-nCov impact questionnaire)	Quarantined HCWs experienced the most distress. Females had more distress compared to males. Older HCWs ≥ 56 years old experienced more distress compared to younger HCWs ≤ 25 years old
Tan et al. (2020) [40] Singapore	COVID-19 Pandemic	N = 470 296 Medical vs. 174 non-medical HCWs (Hospital)	Depression, Anxiety and Stress (DASS-21) Distress (IES-R)	14.5% anxiety, 8.9% depression 6.6% stress 7.7% PTSD symptoms Anxiety and distress were significantly higher among nonmedical HCWs that the medical personnel
Temsah et al. (2020) [41] Saudi Arabia	COVID-19 Pandemic	N= 582 HCW (hospital)	Anxiety (GAD-7) Worry (1–5 worry rating scale)	68.25% mild anxiety, 20.8% moderate anxiety, 2.9% very high anxiety 41.1% were more stressed about COVID than MERS-CoV. The most frequent concern was transmitting the infection to family and friends than to themselves.
Wang et al. (2020) [42] China	COVID-19 Pandemic	N = 123 48 Doctors 75 Nurses (Children’s hospital)	Sleep quality (PSQI) Anxiety (SAS) Depression (SDS)	38% Sleep disturbance 7% Anxiety 25% Depression Sleep disturbance associated factors: Being an only child Exposure to COVID-19 patients Depression
Wu et al. (2020) [43] China	COVID-19 Pandemic	N = 190 HCWs 96 FL (frontline) 94 UW (usual ward)	Burnout (MBI)	The group working on the FLs had a significantly lower frequency of burnout (13% vs. 39%) and were less worried about being infected compared with the UW group. The possible explanation for this unexpected trend was FL HCWs had received timely and accurate information hence they had a high sense of control of their situation
Wu and Wei (2020) [44] China	COVID-19 Pandemic	N = 120 60 cases (COVID designated hospitals) 60 controls (non-COVID designated hospital	Sleep quality (PSQI) Anxiety (SAS) Depression (SDS) General symptoms (SCL-90) PTSD (PCL-C)	Poor sleep quality, anxiety, depression, and general health symptoms were higher among cases (frontline workers in COVID designated hospitals) compared to the controls. Cases had higher levels of anxiety, depression, and insomnia compared to the controls.
Xiao et al. (2020) [45] China	COVID-19 Pandemic	N = 958 HCWs (Hospital)	Anxiety And depression (HAD) Stress (PSS)	55.1% psychological stress 54.2% anxiety 58% depression Stress, Anxiety and Depression levels related to: Female gender Exposure to confirmed cases
Xiaoming et al. (2020) [46] China	COVID-19 Pandemic	N = 8817 HCWs (Hospital)	Depression (PHQ-9) Anxiety (GAD-7) Suicidal and self-harm ideation (SSI)	30.2% Depression, 20.7% Anxiety, 46.2% Somatic symptoms Risk factors of psychological impact: female, single, Tujia minority, low educational background, county hospital, need for psychological assistance, no confidence, ignorance about the epidemic, willingness to attend parties, and poor self-rated health condition
Xing et al. (2020) [47] China	COVID-19 Pandemic	N = 548 HCWs (Hospital)	Mental health status (SCL-90)	The overall mean SCL90 score of somatization, obsessive-compulsive, anxiety, phobic anxiety, and psychoticism was much higher in the HCWS compared to the national general population (norm group)
Zhang et al. (2020) [48] China	COVID-19 Pandemic	N = 1563 HCWs (hospital)	Insomnia (ISI) Anxiety (GAD) Depression (PHQ-9) Psychological response (IES)	36.1% insomnia symptoms Insomnia symptoms associated with: Lower education, Being a doctor, Female sex Currently working in an isolation unit Worried about being infected. Perceived lack of helpfulness Very strong uncertainty regarding Effective disease control
Zhang et al. (2020) Iran [49]	COVID-19 Pandemic	N = 304 HCWs (hospital)	Distress (K6) (SF-12) Depression (PHQ-12)	28.0% Anxiety, 30.6% Depression, 20.1% Distress Older workers better mental but not physical health Females had more distress and depression. HCWs at private institutions had better mental health than those at public institutions. PPE access predicted better physical, mental health, and less distress
Zhu et al. (2020) [50] China	COVID-19 Pandemic	N = 165 HCWs (hospital)	Anxiety (SAS) Depression (SDS) Coping (SCSQ)	Nurses had more Anxiety symptoms compared to doctors (27.9% vs. 11.4%) Risk factors Anxiety—History of depression or anxiety Depression—Female
Alsubaie et al. (2019) [51] Saudi Arabia	MERS-CoV Epidemic	N =516 HCWs (hospital)	Knowledge, anxiety (self-developed questionnaire)	The mean anxiety score was the same for physicians, nurses, and technicians. Non-physicians expressed higher levels of anxiety toward the risk of transmitting MERS-CoV to their families
Park et al. (2018) [52] Korea	MERS-CoV Epidemic	N = 187 Nurses	Overall health status (SF-36) Stigma (self) Stress (PSS-10)	Greater stigma was directly associated with worse mental health. Hardiness was inversely related to mental health via stress
Oh, et al. (2017) [53] South Korea	MERS-CoV Epidemic	N = 313 nurses (hospital)	Stress (stress questionnaire)	The group exposed to MERS confirmed or suspected cases experienced more stress as compared to those who had not exposed to it. Prior outbreak nursing experience had a protective effect
Tang et al. (2017) [54] China	H7N9 Epidemic	N = 102 26 Doctors, 62 Nurses and 14 Interns (Hospital)	PTSD (PCL-C)	20.59% PTSD symptoms Higher scores: Nurses Female Low professional title Frequent contact with patients Aged between 20 years and 30 years. Less than three years of work experience No outbreak training or related experience
Ji et al. (2017) [55] Sierra Leone (SL)	Ebola Epidemic	N =143 59 SL medical staff 21 SL logistic staff, 22 SL medical students, 41 Chinese medical staff, 18 EVD survivors. (hospital)	Psychological Symptoms (SCL-90-R)	The order of psychological symptoms from high to low was EVD survivors, SL medical staff, SL logistic staff, SL medical students, and Chinese medical staff. Psychological symptoms were the highest in EVD survivors and the lowest in Chinese medical staff. Mental state of Chinese medical staff was the same at arrival and before leaving.
Bukhari et al. (2016) [56] Saudi Arabia	MERS-CoV Epidemic	N = 386 HCWs (hospital)	Perception of exposure, perceived risk of infection and distress (IES)	Worry about contracting MERS-CoV: 7.8% extremely worried, 20.5% very worried. Worry about transmitting MERS-CoV to family members: 12.2% extremely worried, 21.0% very worried. Females were more worried than males
Khalid et al. (2016) [57] Saudi Arabia	MERS-CoV Epidemic	N = 117 (Hospital)	Stress and coping strategies (Self-developed questionnaire)	96% were stressed by seeing colleagues contracting the infection, being intubated for respiratory failure, and caring for these sick colleagues. 94% were worried about transmitting MERS-CoV to family and friends. 96% were nervous and scared. Following strict personal protective measures was the most common coping strategy
Kim and Choi (2016) [58] Korea	MERS-CoV Epidemic	N= 215 nurses (Hospital)	Burnout (OLBI) Stress (Parker and DeCotiis) Fear (self-developed scale)	Burnout was higher in those who had nursed MERS-CoV infected or suspected patients than those who did not. Job stress was the biggest influencing factor of burnout. Poor hospital resources for treatment of MERS-CoV and poor support from family and friends increased burnout
Lehmann et al. (2016) [59] Germany	Ebola Epidemic	N = 86 HCWs group1: internal medicine ward group2: ebola treatment ward group3: laboratory (hospital)	Health-related quality of life (SF-12) Anxiety (GAD-7) Depression (PHQ-9)	No significant differences in HrQoL, subjective risk of infection, and most other psychosocial variables. Ebola patient treatment group had higher levels of social isolation than both other groups. The best predictors of poor physical and mental HrQoL were perceived lack of knowledge about the Ebola virus disease and fatigue
Li et al. (2015) [60] Liberia	Ebola Epidemic	N = 52 16 nurses and 13 cleaners (hospital)	Psychological status (SCL90-R) (PST) Distress (PSDI)	Mental distress among participants was not very serious. Cleaners had higher levels of obsessive compulsive, anxiety, positive symptom total and phobic anxiety vs. Treatment staff. Males had more interpersonal sensitivity and paranoid ideation than females
Mohammed et al. (2015) [61] Nigeria	Ebola Virus Disease (EVD) Epidemic	N = 117 (45 HCWs) (community)	Psychological distress (GHQ) Social Support (OSSS)	Non HCWs had higher levels of distress compared to HCWs. Losing a relation to the EVD outbreak was associated with high levels of distress.
Liu et al. (2012) China [62]	SARS Epidemic	N = 549 HCWs (Hospital)	Depression (CES-D) Stress (IES) Trauma exposure (self-developed questionnaire)	Depression: 7.2% Mild, 14.0% Moderate, 8.8%High Being single, females, history of quarantine, history of other traumatic events before SARS, and perceived SARS-related risk level during the outbreak increased the odds of having a high level of depressive symptoms 3 years later. High stress during and after the outbreak was associated with high current depressive symptoms. Altruistic acceptance of risk reduced depressive symptoms
Matsuishi et al. (2012) [63] Japan	H1N1 Pandemic	N = 1625 HCWs (hospital)	Stress (IES)	Workers in high-risk work environments had higher stress and exhaustion than did workers in low-risk work environments. Total stress score of nurses was higher than that of doctors. HCWs in their 50s felt more exhaustion as compared with workers in their 20s
Goulia et al. (2010) [64] Greece	A/H1N1 Pandemic	N = 436 (Hospital)	Anxiety (Self-developed questionnaire) Distress (GHQ-28)	20.7% mild to moderate psychological distress (GHQ-28 > 5) 6.8% severe psychological distress (GHQ-28 > 11) 56.7% moderately high anxiety The most frequent concern was infection of family and friends and the health consequences of the disease. Nurses had highest distress compared to other HCWs
Wu et al. (2009) [65] China	SARS Epidemic	N = 549 (hospital)	Psychological distress (IES) Work exposure, exposure to traumatic events, Fear	10% had post-traumatic symptoms. Altruistic acceptance of risk was negatively related to PTS. High PTS symptoms associated with: History of quarantine, age under 50 years, high levels of exposure to SARS patients, high perceived SARS related risk levels, higher levels of current fear of SARS
Styra et al. (2008) [66] Canada	SARS Epidemic	N = 248 HCWs 88 Low Risk vs. 160 High Risk (hospital)	Self-developed questionnaire	High risk HCWs experienced greater distress Factors that cause distress (a) perception of risk to themselves, (b) impact of SARS crisis on their work life (c) depressive affect (d) working in a high-risk unit (e) HCWs who cared for only one SARS patient experienced more post-traumatic stress symptoms compared to those caring for multiple SARS patients
Wu et al. (2008) [67] China	SARS Epidemic	N = 549 HCWs (hospital)	Depression (CES-D) Alcohol abuse/dependence (NHSDA) Distress (IES-R)	19% of the hospital employees had at least one alcohol use-related symptom, while <5% had two or more symptoms. Alcohol use related symptoms higher in: Male Age between 36 and 50, Low educational levels Upper-middle level family income levels Units with high levels of exposure to SARS Quarantined during the SARS outbreak.
Chen et al. (2007) [68] Taiwan	SARS Epidemic	N = 90 HCWs 82 control subjects (hospital)	General health status (MOS SF-36)	SARS HCWs had low scores vs. control group, for vitality, social functioning, and mental health. The HCWs social functioning, role emotional, and role physical subscales significantly improved after self-quarantine and off-duty shifts.
Lin et al. (2007) [69] Taiwan	SARS Epidemic	N = 92 HCWs (emergency department vs. psychiatry ward) (Hospital)	Psychological status (DTS-C) (CHQ-12)	19.3% had symptoms of PTSD (DTS-C scores >40) 47.78% had minor psychiatric morbidity (CHQ-12 scores >3) Emergency department staff had higher psychiatric morbidity, and experienced PTSD symptoms more often and more severely than psychiatry ward staff.
Marjanovic et al. (2007) [70] Canada	SARS Epidemic	N = 333 nurses (hospital)	Burnout (MBI) Anger (STAXI) Organizational support (SPOS) Trust in equipment/infection control, avoidance, and vigor (self-developed)	Higher levels of vigor, organizational support, and trust in equipment/infection control initiative decreased avoidance behavior, burnout, and state anger. Lower levels of contact with SARS patients, and lesser time spent in quarantine decreased avoidance behavior, burnout, and state anger.
Cheng et al. (2006) Taiwan [71]	SARS Epidemic	N = 116 nurses (hospital)	Anxiety (SAS) Depression (SDS) Sleep quality (PSQI)	Moderate anxiety, Moderate depression, Moderate poor sleep quality
Fiksenbaum et al. (2006) [72] Canada	SARS Epidemic	333 nurses (hospital)	Perceived SARS threat (self-developed questionnaire) Emotional exhaustion (MBI-GS) State anger (STAXI)	Nurses who had contact with SARS patients. - higher levels of perceived SARS threat, - higher levels of emotional exhaustion - Higher levels of state anger Higher levels of organizational support predicted lower perceived SARS threat, emotional exhaustion, and state anger.
Maunder et al. (2006) [73] Canada SARS Epidemic	SARS Epidemic	N = 769 73.5% nurses, 8.3% clerical, 2.9% physicians, 2.3% respiratory therapists, 12.9% others HCWs (hospital)	Stress (IES) Distress (K10) Burnout (MBI) Increase in smoking, drinking alcohol, Stigma, job stress, (WCQ), Toronto HCWs vs. Hamilton HCWs	Toronto hospitals treated SARS patients. Hamilton hospitals did not treat SARS patients. Toronto HCWs reported significantly higher levels of burnout, psychological distress and post-traumatic stress compared to Hamilton HCWs. Toronto HCWs had an increase in smoking and drinking alcohol and other behaviors that can interfere with work and relationship
Chan SSC et al. (2005) [74] Hong Kong	SARS Epidemic	N = 1470 nurses (hospital)	General health status, anxiety, and stress (SARS NSQ)	52.6–63.5% considered their general health to be good. 68.3–80.1% nurses always/often perceived stress from the SARS epidemic. 85.9–95.6% nurses perceived their stress came from work.
Cheng et al. (2005) Taiwan [75]	SARS Epidemic	N = 184 nurses 85 high risk group 30 conscripted from low to high-risk group 69 control group (hospital)	Stress (IES) Psychiatric morbidity (SCL-90-R)	11% had stress reaction syndrome. Of these, 17% high-risk group 10% conscripted group 2% control group High risk group had higher stress and psychiatric morbidity than to the control group. Conscripted group had higher stress and psychiatric morbidity than to the control group and high-risk group.
Grace et al. (2005) [76] Canada	SARS Epidemic	N = 193 physicians (Hospital)	Psychological distress and stigma (Self-designed questionnaire)	Psychological distress: Physicians providing direct care to SARS patients (45.7%) Physicians not providing direct care to SARS patients (17.7%) Stigma 36%
Ho et al. (2005) [77] Hong Kong	SARS Epidemic	N = 179 Sample 1: (N= 82) during peak of epidemic. Sample 2: (N = 97) HCWs who recovered from SARS (hospital)	Fear (SFS) Self-Efficacy (SES) PTSD (IES)	Fear Sample 1: fear related to infection. Sample 2: fear about death, discrimination, quarantine, and side effects of SARS treatment. Self-efficacy Sample 1: low self-efficacy related to more fear. Sample 2: low self-efficacy related to insecurity and instability. PTSD Sample 2: SARS-related fears strongly related to PTSD
Koh et al. (2005) [78] Singapore	SARS Epidemic	N = 10,511 (Hospital)	Stress (IES) Perception of risk and stigma (self-developed questionnaire)	76% perceived a great personal risk of falling ill with SARS. 56% of clinical staff in contact with SARS patients had increased work stress. 53% experienced increase in workload 49% experienced social stigmatization 31% experienced ostracism by family members
Lee et al. (2005) [79] Taiwan	SARS Epidemic	N = 26 nurses (Hospital)	Stress and coping strategies (self-developed SARS team questionnaire)	92% stressed about being negligent and endangering co-workers, 92% stressed about frequent modification of infection control procedures. 92% stressed about the uncertainty of when the epidemic will be under control. 89% stressed about inflicting SARS on family members. Taking protective measures and actively acquiring more information were the most common coping strategies. Adequate PPE and reasonable staffing/shift were motivating factors to work during the outbreaks
Phua et al. (2005) [80] Singapore	SARS Epidemic	N = 96 HCWS (hospital)	Coping (COPE) Psychiatric morbidity (GHQ) Stress (IES)	17.7% psychiatric morbidity (IES ≥26) 18.8% psychiatric morbidity (GHQ ≥ 5) Nurses reported higher psychiatric morbidity compared to physicians.
Tham et al. (2005) [81] China	SARS Epidemic	N = 99 41 doctors 58 nurses (Hospital)	Post event morbidity (IES) Psychiatric morbidity (GHQ)	17.7% Post-traumatic stress morbidity (IES ≥ 26) 18.8% Psychiatric morbidity (GHQ 28 ≥ 5) Nurses had higher IES and psychiatric morbidity than the doctors. Females had higher IES and psychiatric morbidity than the males
Wong et al. (2005) [82] Hong Kong	SARS Epidemic	N = 466 HCWs (Hospital)	Distress (Self-designed questionnaire) Coping strategies (COPE)	Distress level was highest for nurses, followed by doctors and HCA. The overall distress level was related to: Vulnerability/loss of control, Health of self Health of family and others, Changes in work, being isolated. Frequently adopted coping strategies were acceptance, active coping, and positive framing
Bai et al. (2004) [83] Taiwan	SARS Epidemic	N = 338 218 HCWs and 79 administrative personnel (hospital)	Stress (SARS-related stress reactions questionnaire)	5% acute stress disorder 20% felt stigmatized and rejected in their neighborhood. 9% HCWs reported reluctance to work or had considered resignation. Quarantine increased stress. HCWs reported more insomnia, exhaustion, and uncertainty about the frequent modifications to infection control procedures compared to administrative staff
Chan and Huak (2004) [84] Singapore	SARS Epidemic	N = 661 Doctors and nurses (hospital)	Psychiatric caseness (GHQ-28) PTSD (IES)	27% had GHQ-28 score ≥ 5, indicating presence of psychiatric symptoms. 20% had IES scores ≥ 30, indicating the presence of post-traumatic stress disorder (PTSD). Doctors were 1.6 times more likely to experience psychiatric symptoms compared with the nurses. Marital status: Single HCWs were 1.4 times more likely to experience psychiatric symptoms compared with married HCWs.
Chong et al. (2004) [85] Taiwan	SARS Epidemic	N = 1257 (Hospital)	Psychiatric morbidity (CHQ)	Psychiatric morbidity 75.3% Those who were responsible for the care of SARS patients manifested higher rates of psychiatric morbidity. Females had greater psychiatric morbidity than men
Chua et al. (2004) [86] Hong Kong	SARS Epidemic	N = 271 HCWs and N = 342 healthy control subjects	Stress (PSS-10)	Stress levels were raised in both groups (PSS-10 ≥ 18), but there were no group differences. PSS-10 HCWs: 18.6; PSS-10 Controls: 18.3 HCWs had more protective psychological effects vs. controls
Nickell et al. (2004) [87] Canada	SARS Epidemic	N = 2001 HCWs N = 510 GHQ	Stress (GHQ-12)	29% had emotional distress. More nurses experienced emotional distress compared to other professionals. Emotional distress was significantly increased in those HCWs who had part-time employment status. Higher levels of concern for self and family were associated with a higher perception of risk of death from SARS
Poon et al. (2004) [88] Hong Kong	SARS Epidemic	N = 1926 1903 HCWs and 230 administrative staff (controls)	Anxiety (STAI) Burnout (MBI)	Anxiety was significantly higher among those who had contact with SARS patients that those who did not have this contact. Frontline HCWs had significantly higher anxiety and burnout compared to the administrative staff controls. Female nurses experienced more anxiety.
Sim et al. (2004) [89] Singapore	SARS Epidemic	N = 277 21 doctors and 186 nurses (Hospital)	Psychiatric morbidity and post-traumatic stress (Self-designed questionnaire)	20.6% Psychiatric morbidity 9.4% Posttraumatic morbidity Psychiatric morbidity and posttraumatic morbidity were associated with higher scores of coping efforts including self-distraction, behavioral disengagement, social support, venting, planning, and self-blame
Sin SS and Huak CY (2004) [90] Singapore	SARS Epidemic	N = 47 therapists.(Hospital)	Psychiatric distress (GHQ) Stress (IES) Self- developed Questionnaire on ways of coping	23.4% Psychiatric symptoms 12.8% Post-traumatic stress symptoms Support from colleagues, taking precautionary measures, getting clear directives and disease information, support from family and friends were the most common helpful coping strategies. Availability of adequate PPE gave HCWs a sense of control and reduced their stress
Tam et al. (2004) [91] Hong Kong	SARS Epidemic	N = 652 HCWs (Hospital)	Psychiatric morbidity (GHQ)	68% high level of stress. 57% psychological distress. 56.7% psychiatric morbidity High stress risk factors younger age, being a nurse, female, direct care of SARS patients and poorer self-rated physical health condition, inadequate social support.
Verma et al. (2004) [92] Singapore	SARS Epidemic	N = 1050 721 GPs N = 329 TCM (traditional Chinese medicine) (General practice)	Psychological distress (GHQ-28) PTSD (IES-R) Stigma (HIV stigma scale)	More GPs were directly involved in the care of patients with SARS. 14.1% GPs, 6% TCMs had psychological distress (GHQ-28 > 7) More GPs had psychological distress compared to TCM practitioners. The mean score of the GHQ somatic, anxiety, and social dysfunction subscales were higher in GPs as compared to practitioners. GPs experienced more stigma.
Wong et al. (2004) [93] Hong Kong	SARS Epidemic	N = 137 GPs (General Practice)	Anxiety (Self-designed questionnaire)	Significant anxiety was found in family doctors. 75% requested more investigations. 25% over-prescribed antibiotics Young doctors found their quality of life more affected than their older colleagues

Abbreviations: Appendix B.

**Table 2:** A summary of the cohort studies included in this review.

Study (Year)/Country	Disease Outbreak	Participants (Setting), Period of Assessment	Mental Health Outcome Measures (Instrument)	Main Findings
Lee et al. (2018) [4] Korea	MERS-CoV Epidemic	N= 359 HCWs (Hospital) 6 Weeks	Distress (IES-R)	First survey: 64.1% PTSD-like symptoms, 51.5% PTSD Second survey (N = 77 from the high-risk group): 54.5% PTSD-like symptoms, 40.3% PTSD PTSD symptoms were higher in HCWs who performed MERS related tasks.
Lung et al. (2009) [94] Taiwan	SARS Epidemic	N = 127 HCWs (hospital) 8 months	Psychiatric morbidity (CHQ), Personality (EPQ) at the first stage and the CHQ again a year later	Initial assessment (shortly after the SARS epidemic was under control): 17.3% had psychiatric symptoms (CHQ > 3) At follow up (after 1 year): 15.4% had psychiatric symptoms (CHQ > 3) Stress was from job, families, and daily life events. A higher percentage of physicians (35%), compared to nurses (25%), developed psychiatric symptoms
Lancee et al. (2008) [95] Canada	SARS Epidemic	N = 139 103 nurses 15 clerical staff (hospital) One year	Distress (IES) Distress (K-10) Burnout (MBI) (SCID) (CAPS)	30% Lifetime prevalence of psychiatric diagnosis 4% New episode major depression Incidence 2% New-onset PTSD incidence 5% New onset psychiatric disorder incidence New episodes associated with history of psychiatric disorder before the outbreak and less years of healthcare experience. New episodes inversely related to perceived adequacy of training
McAlonan et al. (2007) [96] Hong Kong	SARS Epidemic	Doctors, nurses, and healthcare assistants First sample 106 High risk vs. 70 low risk Follow up. 71 High Risk 113 Low Risk (Hospital) One year	First sample Stress (PSS-10) Follow up sample Depression, Anxiety and Stress (DASS-21) Post-traumatic stress (IES) (PSS-10)	2003 peak of SARS outbreak PSS -10 scores for both groups were elevated but not significantly different from each other. High Risk (17.0) Low risk (15.9) 2004 Follow up. High Risk group remained highly stressed. High risk (18.56) Low risk (14.81) High-Risk group also had higher levels of depression, anxiety, and post-traumatic stress.
Su et al. (2007) [97] Taiwan	SARS Epidemic	N = 102 Nurses 70 SARS 32 Non-SARS (hospital) 7 Weeks	Depression (BDI) Anxiety (STAI) Post-traumatic Stress (DTS-C) Insomnia (PSQI)	Depression symptom ratings decreased as the SARS epidemic decreased regardless of which group (SARS vs. non-SARS unit nurses) was assessed. Anxiety symptoms decreased as a function of time. Fifty percent decrease in PTSD symptom scores at the end of the study for each group. After 7 weeks: Depression, insomnia, and stress was higher in SARS unit nurses vs. non-SARS unit nurses. Depression (38.5% vs. 3.1%) Insomnia (37% vs. 9.7%) Post-traumatic stress symptoms (33% vs. 18.7%) No differences in anxiety

Abbreviations in table of results: Appendix B.

**Table 3:** Critical appraisal of cross-sectional studies.

Study	Johanna Briggs Institute Score	Were the Criteria for Inclusion in the Sample Clearly Defined?	Were the Study Subjects and the Setting Described in Detail?	Exposure Measured in a Valid and Reliable Way?	Objective, Standard Criteria Used for Measurement of the Condition?	Confounding Factors Identified?	Strategies to Deal with Confounding Factors Stated?	Outcomes Measured in a Valid and Reliable Way?	Appropriate Statistical Analysis Used?
Amerio et al. (2020)	8	Y	Y	Y	Y	Y	Y	Y	Y
Cai et al. (2020)	5	Y	Y	Y	Y	N	N	N	Y
Chew et al. (2020)	8	Y	Y	Y	Y	Y	Y	Y	Y
Du et al. (2020)	5	N	Y	Y	Y	N	N	Y	Y
Hacimusalar et al. (2020)	8	Y	Y	Y	Y	Y	Y	Y	Y
Hu et al. (2020)	6	Y	Y	Y	Y	N	N	Y	Y
Kang et al. (2020)	7	N	Y	Y	Y	Y	Y	Y	Y
Lai et al. (2020)	8	Y	Y	Y	Y	Y	Y	Y	Y
Li et al. (2020)	6	Y	Y	Y	Y	N	N	Y	Y
Liang et al. (2020)	5	N	Y	Y	Y	N	N	Y	Y
Liu et al. (2020)	8	Y	Y	Y	Y	Y	Y	Y	Y
Lu et al. (2020)	8	Y	Y	Y	Y	Y	Y	Y	Y
Mo et al. (2020)	5	Y	Y	Y	Y	Y	Y	Y	Y
Qi et al. (2020)	8	Y	Y	Y	Y	Y	Y	Y	Y
Que et al. (2020)	8	Y	Y	Y	Y	Y	Y	Y	Y
Shechter et al. (2020)	6	Y	Y	Y	Y	N	N	Y	Y
Sun et al. (2020)	8	Y	Y	Y	Y	Y	Y	Y	Y
Tan et al. (2020)	7	N	Y	Y	Y	Y	Y	Y	Y
Temsah et al. (2020)	8	Y	Y	Y	Y	Y	Y	Y	Y
Wang et al. (2020)	7	N	Y	Y	Y	Y	Y	Y	Y
Wu et al. (2020)	8	Y	Y	Y	Y	Y	Y	Y	Y
Wu and Wei (2020)	8	Y	Y	Y	Y	Y	Y	Y	Y
Xiao et al. (2020)	7	N	Y	Y	Y	Y	Y	Y	Y
Xiaoming (2020)	8	Y	Y	Y	Y	Y	Y	Y	Y
Xing et al. (2020)	8	Y	Y	Y	Y	Y	Y	Y	Y
Zhang et al. (2020)	8	Y	Y	Y	Y	Y	Y	Y	Y
Zhang et al. (2020)	5	N	Y	Y	Y	N	N	Y	Y
Zhu et al. (2020)	8	Y	Y	Y	Y	Y	Y	Y	Y
Alsubaie et al. (2019)	6	N	Y	Y	N	Y	Y	Y	Y
Park et al. (2018)	8	Y	Y	Y	Y	Y	Y	Y	Y
Oh, et al. (2017)	8	Y	Y	Y	Y	Y	Y	Y	Y
Tang et al. (2017)	6	Y	Y	Y	Y	N	N	Y	Y
Ji et al. (2017)	6	N	Y	Y	Y	N	N	Y	Y
Bukhari et al. (2016)	6	N	Y	Y	Y	Y	N	Y	Y
Khalid et al. (2016)	5	Y	Y	Y	Y	N	N	Y	N
Kim et al. (2016)	8	Y	Y	Y	Y	Y	Y	Y	Y
Lehmann et al. (2016)	8	Y	Y	Y	Y	Y	Y	Y	Y
Li et al. (2015)	8	Y	Y	Y	Y	Y	Y	Y	Y
Mohammed (2015)	8	Y	Y	Y	Y	Y	Y	Y	Y
Liu et al. (2012)	8	Y	Y	Y	Y	Y	Y	Y	Y
Matsuishi et al. (2012)	8	Y	Y	Y	Y	Y	Y	Y	Y
Goulia et al. (2010)	8	Y	Y	Y	Y	Y	Y	Y	Y
Wu et al. (2009)	8	Y	Y	Y	Y	Y	Y	Y	Y
Styra et al. (2008)	8	Y	Y	Y	Y	Y	Y	Y	Y
Wu et al. (2008)	8	Y	Y	Y	Y	Y	Y	Y	Y
Chen (2007)	8	Y	Y	Y	Y	Y	Y	Y	Y
Lin et al. (2007)	6	Y	Y	Y	Y	N	N	Y	Y
Marjanovic et al. (2007)	6	Y	Y	Y	Y	N	N	Y	Y
Chen et al. (2006)	8	Y	Y	Y	Y	Y	Y	Y	Y
Fiksenbaum et al. (2006)	8	Y	Y	Y	Y	Y	Y	Y	Y
Maunder et al. (2006)	8	Y	Y	Y	Y	Y	Y	Y	Y
Chan et al. (2005)	8	Y	Y	Y	Y	Y	Y	Y	Y
Cheng et al. (2005)	8	Y	Y	Y	Y	Y	Y	Y	Y
Grace et al. (2005)	5	Y	Y	Y	N	N	N	Y	Y
Ho et al. (2005)	8	Y	Y	Y	Y	Y	Y	Y	Y
Koh et al. (2005)	8	Y	Y	Y	Y	Y	Y	Y	Y
Lee et al. (2005)	5	N	Y	Y	Y	N	N	Y	Y
Phua et al. (2005)	6	Y	Y	Y	Y	N	N	Y	Y
Tham et al. (2005)	8	Y	Y	Y	Y	Y	Y	Y	Y
Wong et al. (2005)	6	Y	Y	Y	Y	N	N	Y	Y
Bai et al. (2004)	6	N	Y	Y	Y	Y	Y	N	Y
Chan et al. (2004)	8	Y	Y	Y	Y	Y	Y	Y	Y
Chong et al. (2004)	8	Y	Y	Y	Y	Y	Y	Y	Y
Chua et al. (2004)	6	Y	Y	Y	Y	N	N	Y	Y
Nickell et al. (2004)	8	Y	Y	Y	Y	Y	Y	Y	Y
Poon et al. (2004)	6	Y	Y	Y	Y	N	N	Y	Y
Sim et al. (2004)	7	N	Y	Y	Y	Y	Y	Y	Y
Sin.S.S. and Huak C.Y (2004)	6	Y	Y	Y	Y	N	N	Y	Y
Tam et al. (2004)	8	Y	Y	Y	Y	Y	Y	Y	Y
Verma et al. (2004)	8	Y	Y	Y	Y	Y	Y	Y	Y
Wong et al. (2004)	5	Y	Y	Y	Y	N	N	N	Y

**Table 4:** Critical appraisal of cohort studies.

Study	Johanna Briggs Institute Score	Were the Criteria for Inclusion in the Sample Clearly Defined?	Were the Study Subjects and the Setting Described in Detail?	Exposure Measured in a Valid and Reliable Way?	Objective, Standard Criteria Used for Measurement of the Condition?	Confounding Factors Identified?	Strategies to Deal with Confounding Factors Stated?	Outcomes Measured in a Valid and Reliable Way?	Appropriate Statistical Analysis Used?	Was the Follow Up Time Reported and Sufficient to Be Long Enough for Outcomes to Occur?	Was Follow Up Complete, and If Not, Were the Reasons to Loss to Follow Up Described and Explored?	Were Strategies to Address Incomplete Follow-Up Utilized?
Lee et al. (2018)	7	Y	Y	Y	Y	N	N	Y	Y	Y	N	N
Lung et al. (2009)	8	N	N	Y	Y	Y	Y	Y	Y	Y	Y	N/A
Lancee et al. (2008)	9	Y	Y	Y	Y	Y	Y	Y	Y	Y	N	N
McAlonan et al. (2007)	9	Y	Y	Y	Y	Y	Y	Y	Y	Y	N	N
Su T.P. (2007)	10	Y	Y	Y	Y	Y	Y	Y	Y	Y	Y	N/A

## Data Availability

The data presented in this study are available at (online version link).

## Appendix A. Search Strategy

The search was performed from May 2020 to end-June 2020. An English language limit was applied. No restrictions were placed on the publication date and location of study. The search terms were grouped into three categories:

Category 1: Population (“healthcare professional”, “healthcare workers”, physician, doctor, nurse)

Category 2: Exposure (epidemic, pandemic)

Category 3: Outcomes (“mental health”, “mental disorder”, psychological, depression, anxiety, stress, burden, insomnia, “sleep disturbance”, burnout, fear, stigma, discrimination).

Mesh terms and synonyms of the keywords were identified and used in the search.

**Table A1:** PubMed Search.

Search	Query	Items Found
#1	(“health personnel” OR “ healthcare provider*” OR “healthcare worker*” OR “healthcare personnel” OR “ healthcare professional*” OR “healthcare staff” OR doctor OR physician OR “physician assistant*” OR nurse OR “healthcare assistant*” OR “allied health*” OR clinician OR “hospital worker*” OR “hospital staff” OR “hospital employee*”)	1,923,975
#2	(epidemic* OR pandemic* OR SARS OR “severe acute respiratory syndrome” OR coronavirus OR MERS OR “middle east respiratory syndrome” OR MERS-CoV OR Ebola OR EVD OR H1N1 OR “influenza type A virus” OR H7N9 OR covid-19 OR 2019-nCoV OR SARS-COV-2 OR “2019 novel coronavirus”)	220,091
#3	mental* OR psychiatric* OR psychological* OR resilience OR depression OR emotio* OR anxiety* OR nervous* OR stress* OR PTSD OR “post-traumatic stress disorder” OR insomnia OR “sleep disorder” OR DIMS OR “ disorder of initiating and maintaining sleep” OR burnout OR exhaustion OR fear OR panic OR stigma* OR discrimination OR “mental health”	3,376,683
#4	#1 AND #2 AND #3	3311

**Table A2:** PsycArticles Search.

Search	Query	Items Found
#1	(“health personnel” OR “ healthcare provider*” OR “healthcare worker*” OR “healthcare personnel” OR “ healthcare professional*” OR “healthcare staff” OR doctor OR physician OR “physician assistant*” OR nurse OR “healthcare assistant*” OR “allied health*” OR clinician OR “hospital worker*” OR “hospital staff” OR “hospital employee*”)	17,759
#2	(epidemic* OR pandemic* OR SARS OR “severe acute respiratory syndrome” OR coronavirus OR MERS OR “middle east respiratory syndrome” OR MERS-CoV OR Ebola OR EVD OR H1N1 OR “influenza type A virus” OR H7N9 OR covid-19 OR 2019-nCoV OR SARS-COV-2 OR “2019 novel coronavirus”)	932
#3	mental* OR psychiatric* OR psychological* OR resilience OR depression OR emotio* OR anxiety* OR nervous* OR stress* OR PTSD OR “post-traumatic stress disorder” OR insomnia OR “sleep disorder” OR DIMS OR “ disorder of initiating and maintaining sleep” OR burnout OR exhaustion OR fear OR panic OR stigma* OR discrimination OR “mental health”	158,189
#4	#1 AND #2 AND #3	117

**Table A3:** PsycInfo Search.

#1	(“health personnel” OR “ healthcare provider*” OR “healthcare worker*” OR “healthcare personnel” OR “ healthcare professional*” OR “healthcare staff” OR doctor OR physician OR “physician assistant*” OR nurse OR “healthcare assistant*” OR “allied health*” OR clinician OR “hospital worker*” OR “hospital staff” OR “hospital employee*”)	344,711
#2	epidemic* OR pandemic* OR SARS OR “severe acute respiratory syndrome” OR coronavirus OR MERS OR “middle east respiratory syndrome” OR MERS-CoV OR Ebola OR EVD OR H1N1 OR “influenza type A virus” OR H7N9 OR covid-19 OR 2019-nCoV OR SARS-COV-2 OR “2019 novel coronavirus”	41,531
#3	mental* OR psychiatric* OR psychological* OR resilience OR depression OR emotio* OR anxiety* OR nervous* OR stress* OR PTSD OR “post-traumatic stress disorder” OR insomnia OR “sleep disorder” OR DIMS OR “ disorder of initiating and maintaining sleep” OR burnout OR exhaustion OR fear OR panic OR stigma* OR discrimination OR “mental health”	2,335,979
#4	#1 AND #2 AND #3	2288

## Appendix B. Abbreviations in Table of Results

AIS Athens Insomnia Scale, BAI Beck Anxiety Inventory, BDI-II Beck Depression Inventory II, BHS Beck Hopelessness Scale, CAPS Clinician-Administered PTSD Scale, CES-D Centre for Epidemiologic Studies Depression Scale, CHQ Chinese health Questionnaire, CHQ-12 Chinese Health Questionnaire-12, COPE Coping Orientation to Problems Experienced, DASS-21 Depression, Anxiety and Stress Scale-21, DRS-15 Dispositional Resilience Scale-15, DTS-C Davidson Trauma Scale-Chinese version, ECR-R Experiences in Close Relationships-Revised, EPQ Eysenck Personality Questionnaire, FS-HPs Fear Scale for Healthcare Professionals, GAD-7 Generalized Anxiety Disorder-7, GHQ-28 General health Questionnaire -28, HAM-A Hamilton Anxiety Score, HAMD Hamilton Depression Scale, HADS Hospital Anxiety and Depression Scale, IES-R Impact Events Scale Revised, ISI -7 Insomnia severity index-7, K-10 Kessler Psychological Distress Scale-10, K-6 Kessler Psychological Distress Scale-6, MBI Maslach Burnout Inventory, MOS SF-36 Medical Outcome Study Short-Form 36 Survey, NHSDA National Household Survey on Drug Abuse, NRS Numeric Rating Scale, OLBI Oldenburg Burnout Inventory, OSSS Oslo Social Support Scale, PCL-C PTSD Checklist-Civilian Version, PC-PTSD Primary Care PTSD screen, PHQ-12 Patient Health Questionnaire-12, PHQ-9 Patient Health Questionnaire-9, PSDI Positive Symptom Distress Index, PSQI Pittsburgh Sleep Quality Index, PSS Perceived Stress Scale, PSS-10 perceived stress scale-10, SARS NSQ SARS Nurses’ Survey Questionnaire, SAS Self-Rating Anxiety Scale, SCID Structured Clinical Interview for DSM-IV, SCL-90 The 90-item symptom checklist, SCSQ Simplified coping style questionnaire, SDS Self-Rating Depression Scale, SES Self-Efficacy Scale, SF-12 Short Form Health Survey-12, SF-36 Short Form Health Survey-36, SFS SARS Fear Scale, SRSR SARS-Related Stress Reactions questionnaire, SSI Suicidal and self-harm ideation, SOS Stress Overload Scale, SPOS Survey of Perceived Organizational Support, SRQ-20 WHO Self-Reporting Questionnaire, STAI The State-Trait Anxiety Inventory, STAXI State-Trait Anger Expression Inventory, TCSQ Trait Coping Style Questionnaire, VAS Visual Analogue Scale, WCQ Ways of Coping Questionnaire, HCW Health Care Worker, HR High Risk, LR Low Risk SL Sierra Leonne, FMW Frontline Medical Workers, GP General Practitioner, TCM Traditional Chinese medicine, SARS Severe Acute Respiratory Syndrome, MERS-CoV Middle East Respiratory Syndrome Coronavirus, COVID Coronavirus Disease A/H1N1 Influenza A Subtype H1N1, EBV Ebolavirus Disease, HCA Healthcare Assistant, FL Frontline, UW Usual wards, PPE Personal protective equipment, PTSD Post-Traumatic Stress Disorder.
